# Evaluation of the effects of COVID-19 on semen parameters and male infertility

**DOI:** 10.5935/1518-0557.20230067

**Published:** 2024

**Authors:** Ana Lívia Mota Araújo, Valéria Lamounier Lellis de Almeida, Thayla Mylena Lopes Costa, Ana Costa Guimarães Mendonça, Maria Lecticia Firpe Penna, Adriana dos Santos, Mariana Gontijo Ramos

**Affiliations:** 1 Faculdade de Ciências Humanas, Sociais e da Saúde - Universidade Fumec. Belo Horizonte, MG, Brazil

**Keywords:** COVID-19, SARS-CoV-2, male infertility, semen parameters

## Abstract

The new coronavirus pandemic resulted in millions of deaths in Brazil and around the world, and presented substantial challenges to society. The shortand long-term clinical manifestations tied to COVID-19 are still poorly understood, and may involve several organs and systems, including the male genital tract, which may lead to impaired fertility. The present study aimed to analyze, through an integrative literature review of articles available in databases, the effects of COVID-19 on parameters related to human semen quality. The analyzed studies reported significant decreases in sperm motility and morphology related to COVID-19. Reductions in concentration and volume were also observed. Inflammatory response is one of the leading mechanisms that may potentially explain the observed changes, although others may also be involved. More studies are needed to better understand the effects, modes of action, as well as other aspects involved in this complex phenomenon.

## INTRODUCTION

Infertility is a reproductive system disorder affecting up to 15% of reproductive-age couples worldwide (Ring *et al.*, 2016). Male-related dysfunctions account for 40 to 50% of all cases of infertility (Farsimadan & Motamedifar, 2020). There are multiple and complex causes and pathological agents involved in infertility; viruses might play an important role in this process, interfering with male reproductive functions (Hezavehei *et al.,* 2021).

*The new coronavirus (SARS-CoV-2) was first isolated in humans in Wuhan province, China,* in December 2019, and described as the agent causing COVID-19, a condition that severely affects human health. An outbreak ensued and in February 2020 the World Health Organization declared it a pandemic (Jebril, 2020). Many deaths have since been confirmed due to complications arising from the disease.

Patients with COVID-19 present symptoms including headache, asthenia, anosmia, fatigue, dry cough, fever and dyspnea (Wong *et al.*, 2020). Studies concerning complications following coronavirus infection have shown that it is a multisystemic disease. Men seem to be more severely affected by SARS-CoV-2 infection, and the causes for this observation are still not well known (Dutta & Sengupta, 2021).

Many studies have been conducted to describe the structure of SARS-CoV-2 and its effects in humans. The virus uses its surface Spike (S) protein to bind to angiotensin converting enzyme II (ACE2) and enter host cells. It also uses cellular serine protease TMPRSS2, allowing the initiation of viral protein S (Hoffmann *et al.*, 2020). Male reproductive system cells, such as spermatogonia and testicle Leydig and Sertoli cells, also express ACE2 (Wang & Xu, 2020).

It has been speculated that SARS-CoV-2 might impair testicular histology, causing morphological and functional changes in these cells. Studies also showed that inflammation induced by coronavirus infection results in orchitis and epididymitis (Chen *et al.*, 2021). *In turn, these conditions might cause male sexual dysfunction and infertility (Li et al., 2020a).*

*Studies* have also demonstrated that the male reproductive system might be vulnerable to SARS-CoV-2 infection. Testicle injuries caused by the virus could cause circulatory changes, reduced number of sperm-producing cells and hormonal changes (Tian & Zhou, 2021). Infection can lead to morphological and functional changes in semen parameters and impaired fertility (Yang *et al.*, 2020; Ma *et al.*, 2021).

It is important to consider that sperm quality in semen is a crucial factor in successful reproduction. The shortand long-term effects of COVID-19, especially on the male reproductive system and semen parameters, are not well known and still challenge the medical scientific community.

Based on the above, the aim of the present literature review looked into the effects of SARS-CoV-2 infection on different semen parameters.

## MATERIALS AND METHODS

The present study presents a literature review on the impacts of COVID-19 on male fertility and sperm quality parameters in particular.

Two independent reviewers performed a search on PubMed/MEDLINE. The search terms were “COVID 19” and “male infertility”. The two reviewers analyzed the studies by title, abstract and keywords and the decision about which studies would be included was made after they were read in full. Thirteen articles were selected and the results are analyzed in the present review.

## RESULTS

The present study describes the effects of COVID-19 on semen motility, concentration, volume and morphology. The main findings of the articles included in this review are demonstrated on Table 1.

**Table 1:** Main findings in semen parameters reported in the included studies.

Study	Semen volume	Sperm concentration	Sperm motility	Sperm morphology
Apaydin *et al*., 2022	No alterations were found.	Reduced in three of seven patients.	Progressive motility reduced in three of seven patients.	Not evaluated
Best *et al.*, 2021	No alterations were found.	Reduced in patients with COVID-19 compared to controls.	Not evaluated	Not evaluated
Donders *et al.*, 2022	No alterations were found	Reduced in 25% of patients. Great decrease relative to early infection.	Reduced in 44% of patients. Great reduction relative to early and severe infection.	Reduced in 67% of patients. Great reduction depending on severity of disease.
Erbay *et al.*, 2021	Reduced when compared to same patients before moderate COVID-19	Reduced when compared to same patients before moderate COVID-19.	Reduced when compared to same patients before moderate COVID-19.	Not evaluated.
Guo *et al*., 2021	No alterations were found.	No alterations were found.	Reduced when compared to controls.	No alterations were found.
Holtmann *et al*., 2020	No alterations were found	Reduced in group of patients with moderate infection compared to those with mild infection and controls.	Reduced in group of patients with moderate infection compared to those with mild infection and controls.	Not evaluated.
Koç & Keseroglu, 2021	Reduced when compared to same patients before COVID-19.	No alterations were found.	Reduced when compared to same patients before COVID-19.	Reduced when compared to same patients before COVID-19.
Li *et al.*, 2020b	Not evaluated.	Reduced in patient with COVID-19 compared to controls.	Not evaluated.	Not evaluated.
Hajizadeh Maleki & Tartibian, 2021	Reduced in patients with COVID-19 compared to controls. No change after disease recovery.	Reduced in patient with COVID-19 compared to controls. Increased after 60 days of recovery.	Reduced in patient with COVID-19 compared to controls. Increased after 60 days recovery.	Reduced in patient with COVID-19 compared to controls. No change after disease recovery.
Rafiee & Bagher Tabei, 2021	Reduced immediately after COVID-19.	Reduced immediately after COVID-19.	Reduced immediately after COVID-19.	Reduced immediately after COVID-19.
Ruan *et al*., 2021	Within WHO lower limits.	Within WHO lower limits.	Within WHO lower limits.	Within WHO lower limits.
Seckin *et al*., 2022	No alterations were found.	No alterations were found.	Patient with 0% motility in the first evaluation after COVID-19 and improvement after three months of recovery, reaching 72.5% progressive motility.	Patient demonstrated 9% of normal morphology after three months recovery.
Temiz *et al*., 2021	No alterations were found.	No alterations were found.	No alterations were found.	Significantly altered in patients with COVID-19 compared to controls.

### Sperm motility

The results revealed a significant association between SARS-CoV-2 infection and decreased sperm motility. Studies comparing males recovered from COVID-19 against healthy males (control group) found significantly decreased sperm motility in individuals with the disease (Ruan *et al.*, 2021; Hajizadeh Maleki & Tartibian, 2021; Holtmann *et al.,* 2020; Guo *et al.,* 2021).

Holtmann *et al*. (2020) divided patients with mild and moderate COVID-19 into different groups and observed lower progressive motility (20%) in the group with mild disease versus patients with moderate involvement (46.1%), suggesting that disease severity might relate to intensity of sperm alteration.

Erbay *et al.* (2021) and Koç & Keseroğlu (2021) followed the same patients in distinct periods of time, before and after they became infected by SARS-CoV-2, and found a significant decrease in both total and progressive sperm motility after infection. Koç & Keseroğlu (2021) also described a significant increase in immotile spermatozoa percentage after COVID-19 diagnosis. Apaydin *et al.* (2022) analyzed a series of seven patients and found that 42.8% (3/7) had decreased progressive sperm motility when evaluated six months after COVID-19, showing that the effects might last for some time.

However, Donders *et al.* (2022), in a more consistent study with 118 patients analyzed at different times after COVID-19 showed different results. A higher percentage (60%) of patients one month after recovering from COVID-19 had decreased motility, according to WHO parameters. When sperm was obtained from patients recovered from COVID-19 for one to two months, the number decreased to 37.2% and to 27.6% for individuals tested more than two months after infection. Hajizadeh Maleki & Tartibian (2021) observed similar results in their study.

In a case report, Seckin *et al*. (2022) described a patient who had normal sperm motility before COVID-19 (86%). Soon after recovery, his first semen analysis showed 0% motility, and three months after it had increased to 44%. This study showed a case of transient asthenozoospermia after SARS-CoV-2 infection, suggesting that changes in sperm motility could be temporary.

Some studies evaluated a possible relationship between fever and decreased motility induced by COVID-19. According to Holtmann *et al*. (2020), patients that tested positive for COVID-19 and had fever during infection presented significantly lower sperm motility compared to patients without fever, suggesting a negative impact of fever on semen quality. However, Erbay *et al*. (2021) evaluated patients with and without fever and found that sperm motility was reduced in both groups.

Rafiee & Bagher Tabei (2021) studied patients before SARS-CoV-2 infection, soon after and three months following disease recovery (a control group and a group treated with N-acetyl cysteine supplement). They observed a significant decrease in sperm motility immediately after infection, which improved three months after recovery in the supplement-treated group, suggesting it might help in fertility recovery following infection.

### Sperm concentration

Two types of results were observed in the included studies: patients with reduced sperm concentration considered infertile according to WHO parameters (lower than 15 million per mL); and patients with reduced concentration at levels above WHO parameters for infertility.

Li *et al*. (2020b) compared semen from men with SARS-CoV-2 infection, with semen from healthy control men, and found reduced sperm concentration on patients with disease. Best *et al*. (2021) and Apaydin *et al*. (2022) compared men recovered from COVID-19 with fertile men and also observed reduced sperm concentration in individuals recovered from the disease.

Donders *et al*. (2022) demonstrated that sperm counts lower than 15 million/mL were six times more frequent in men tested one month after COVID-19 (37%) than in men tested two months after infection (6.3%), suggesting this parameter might normalize after recovery from disease.

Guo *et al*. (2021) and Hajizadeh Maleki & Tartibian (2021) observed a significant decrease in sperm concentration in patients recovered from COVID-19 when compared to controls. Ruan *et al*. (2021) showed a reduction in sperm concentration in patients with severe disease compared to those with mild or moderate involvement. Holtmann *et al*. (2020) also detected decreased concentrations in individuals with moderate disease and in patients with fever. It is then possible to suggest that sperm concentration was significantly lower in men that contracted COVID-19 when compared to healthy controls, and that these alterations might impact male fertility. However, long term effects need to be deeply evaluated.

Rafiee & Bagher Tabei (2021) showed that men with COVID-19 had a significantly worse sperm quality parameters compared to parameters prior to infection. They treated a group of these patients with N-acetyl cysteine (NAC), a derivative of amino acid L-cysteine, used mainly as an antioxidant supplement, and observed that NAC intake significantly improved sperm total motility, sperm morphology, and sperm concentration. They suggested that NAC could be used to improve sperm parameters following COVID-19.

### Semen volume

Volume was the least affected parameter of all semen quality parameters analyzed in our study.

Temiz *et al*. (2021), Ruan *et al*. (2021) and Best *et al*. (2021) found normal semen volumes in COVID-19 patients and controls, without significant differences between them. However, Holtmann *et al*. (2020) and Hajizadeh Maleki & Tartibian (2021) detected decreased semen volumes in samples from COVID-19 patients when compared to controls, although the values were above the WHO fertility thresholds.

Apaydin *et al*. (2022) showed that two of seven patients with altered spermograms six months after COVID-19 had semen volumes below 1.5mL. Guo *et al*. (2021) showed that only 9.8% (4/41) of the samples from patients recovered from the disease had semen volumes of less than 1.5 mL, suggesting that there was no difference in this parameter relative to controls. Other studies also detected differences in semen volumes following COVID-19. However, despite the decreases in these values, they were within the normal range according to the WHO (Erbay *et al*., 2021; Koç & Keseroğlu, 2021).

### Sperm morphology

When sperm morphology was evaluated, important findings were described in the studies analyzed in the present review.

Donders *et al.* (2022) analyzed the semen from 118 individuals recovered from COVID-19 after one, two and between two and six months after infection, and suggested that of all parameters, morphology was the most severely affected. According to the study, 67% of the patients had teratozoospermia. They also described that, differently from other semen quality parameters, morphology was not apparently normalized after recovery from COVID-19, that is, the values remained low even six months after infection.

Studies by Temiz *et al.* (2021) and Hajizadeh Maleki & Tartibian (2021) in men recovered from COVID-19 demonstrated that morphology was one of the most affected semen parameters when compared to controls. Likewise, Hajizadeh Maleki & Tartibian (2021) followed patients for 10, 20, 30, 40, 50 and 60 days after COVID-19 and found no improvement in morphology during this period of time. However, Ruan *et al.* (2021)*studied men 25, 50, 75 and 90 days after disease recovery and noted that morphology parameters remained within the normal range according to the WHO and found an increased percentage of sperm with normal morphology during recovery from COVID-19.*

When the same patients were compared before and after COVID-19, sperm morphology was significantly affected, but remained normal according to WHO parameters (Koç & Keseroğlu, 2021). Semen analysis from 200 men before COVID-19 and two and three months after infection resolution showed lower proportions of normal sperm morphology parameters within the first two months from infection and improved parameters in late follow-up (Rafiee & Bagher Tabei, 2021).

Differently from other authors, Guo *et al.* (2021) analyzed semen obtained from infected men (around 76 days after infection) and found no differences between individuals with disease and healthy controls, suggesting that sperm morphology was not affected by COVID-19.

## DISCUSSION

SARS-CoV-2 and ACE receptor in spermatogenesis

Prior research demonstrated that angiotensin-converting enzyme 2 can be expressed in different extrapulmonary tissues, including the testicles (Donoghue *et al.*, 2000; *Tipnis et al., 2000). Impaired spermatogenesis in men with lower levels of ACE2, compared to* fertile individuals, suggests that this enzyme might play an important role in steroidogenesis regulation, hence affecting germ cells and reproductive health (*Reis et al.,* 2010; Pan *et al.,* 2013).

SARS-CoV-2 uses the ACE2 receptor to establish infection in host cells (Rothan & Byrareddy, 2020). The male reproductive tract might be directly vulnerable to coronavirus invasion via virus binding to the enzyme present in testicular cells, leading to reduced spermatogenesis and fertility problems (Fraietta *et al*., 2020).

A recent study by Valdivia *et al.* (2020) detected expression of the MAS receptor in mature human spermatozoa and observed that its activation modulated sperm motility. This receptor is important in RAS (renin-angiotensin system) cell signaling mediated by ACE2/Ang-(1-7) (angiotensin-(1-7))/MAS receptor axis, expressed by testicular cells (Reis *et al.*, 2010). ACE2 is important in sperm biology, as MAS activation by the enzyme leads to a phosphorylation cascade that inhibits intrinsic apoptotic pathways in spermatozoa, keeping them in a viable mobile state (Aitken, 2021; Koppers *et al*., 2011).

In the present study, SARS-CoV-2 infection was related to reduced sperm mobility, and it is reasonable to suggest that these findings might be partly due to virus interference with the described cell signaling pathways.

### The immune system in SARS-CoV-2 infection

Viral induced immune inflammatory response might be another possible mechanism to suggest the effects of testicular damage and consequent semen alterations caused by COVID-19, with increased cytokine production, leukocyte infiltration and related hormonal changes.

Immune response is known to play a role against many infectious agents, especially viruses. In COVID-19, a balanced inflammatory response is associated with favorable outcomes and better recovery from the disease (Brandão *et al*., 2020). On the other hand, patients with an exacerbated immune response to SARS-CoV-2 are likely to develop more severe forms of the disease, corroborated by other associated stress conditions and/or comorbidities (Zhou *et al.*, 2020).

The cytokine storm that develops in severe cases of COVID-19 is a result of immune response hyperactivation, which affects multiple organs. There is a correlation between increased proinflammatory cytokines, such as tumor necrosis factor (TNF) and interleukin-6 (IL-6), and heart, liver and kidney failure. Intense inflammatory response, potentially associated with fever, might be an important factor in testicular involvement and alterations in semen quality described in the analyzed studies (Abdelhamid *et al.*, 2023).

It is important to emphasize, however, that the different methodologies used in the studies, including patients recovered from COVID-19 given different treatment protocols, may have affected some immune parameters. These are complex questions that require more studies to be better elucidated.

### Fever and semen alterations

Fever, depending on its duration and degree, has a negative impact on sperm quality. Acute fever negatively affects sperm parameters, reducing concentration and increasing the presence of abnormal sperm forms (Andrade-Rocha, 2013). Holtmann *et al*. (2020) demonstrated that patients with fever presented reduced sperm motility, concentration and volume compared to individuals without fever. Erbay *et al*. (2021) analyzed patients with and without fever and found that sperm parameters were reduced in both groups, indicating that fever might not be the only element associated with these specific alterations.

It is important to consider fever as one of the symptoms in COVID-19 that might affect sperm parameters. Physicians must counsel patients about the risk of damage to testicular cells and sperm parameters, especially individuals with severe forms of the disease (Abdelhamid *et al*., 2023). However, this subject is still controversial and more controlled studies are required to further examine and understand the complex mechanisms involved in fever during and after recovery from SARS-CoV-2 infection.

## CONCLUSION

The coronavirus pandemic is still a relevant public health concern, with many unknown aspects related to pathogenesis, including short and long terms effects in different organs and systems, and the male genital tract in particular.

The present study demonstrated that SARS-CoV-2 infection caused significantly decreases in sperm quality parameters, especially sperm motility, and altered sperm morphology.

A limitation to the study was the many different methodologies used in the included studies, with some comparing patients to controls, others the data from the same patients before and after COVID-19, and some following patients during different periods of disease recovery. Other studies evaluating the long term impacts of COVID-19 on semen quality are needed.

The impact of COVID-19 on male fertility and its mechanisms are still vague in some aspects, as scientific research is recent, and more well-designed studies concerning the evaluation of sperm quality, reproduction and male fertility are required to further address and understand the many complex, challenging and interesting phenomena involved iin this subject.
